# Editorial: Special Issue “Liquid Crystals II”

**DOI:** 10.3390/molecules30061373

**Published:** 2025-03-19

**Authors:** Viorel Cîrcu, Doina Manaila-Maximean, Valery A. Loiko

**Affiliations:** 1 Department of Inorganic and Organic Chemistry, Biochemistry and Catalysis, University of Bucharest, Boulevard Regina Elisabeta Nr. 4-12, 030018 Bucharest, Romania; 2 Department of Physics, National University of Science and Technology Politehnica Bucharest, 313 Spl. Independentei, 060042 Bucharest, Romania; 3 Academy of Romanian Scientists, 3 Ilfov, 050044 Bucharest, Romania; 4 B.I. Stepanov Institute of Physics, National Academy of Sciences of Belarus, 68-2 Niezalezhnastsi Avenue, 220072 Minsk, Belarus; loiko@ifanbel.bas-net.by

## 1. Introduction

The Special Issues of the Multidisciplinary Digital Publishing Institute (MDPI) are well-known and highly appreciated in the scientific community. The MDPI editorial process (https://www.mdpi.com/editorial_process, accessed on 17 March 2025), involves managing editors, appropriate Academic Editors and Guest Editors. In cases of conflicts of interest (for example when one of the papers has a Guest Editor as author or co-author), in the Special Issue review process an Editorial Board Member is involved. Special Issues have a start date and a deadline for manuscript submissions. Articles are published immediately after they have been accepted, so one cannot draw conclusions about the number of articles published in a Special Issue during this activity and time interval.

Due to attracted interest, authors and editors often request an extension of the submission period. Also, the themes of the Special Issues and Guest Editors who were successful are often part of subsequent editions. The same applies to the Special Issue *Liquid Crystals II*, which succeeds the Special Issue *Liquid Crystals*, 2020 [1], which has the same Guest Editors.

Liquid crystals (LCs) represent a unique state of matter with properties that bridge solids and liquids, offering a vast range of applications in display technologies, sensors, and optoelectronic devices [2]. Over the past few decades, research into LCs has evolved from fundamental studies of phase behavior to sophisticated molecular engineering that enables precise control over their physical properties. In particular, metallomesogens, liquid crystal dimers, cholesteric liquid crystals, and chiral smectic esters have emerged as key materials with tunable functionalities. The integration of metal ions or chirality into liquid crystals, along with the design of complex molecular architectures have led to remarkable advancements in electro-optical modulation, ionic conductivity, and the luminescence behavior of new materials. This editorial highlights recent breakthroughs in liquid crystalline materials, emphasizing their structural innovations, dynamic behaviors, and application-driven functionalities.

## 2. An Overview of the Published Articles

The Special Issue *Liquid Crystals II* is a collection of eight research papers that address recent progress in both experimental and theoretical aspects of liquid crystals science and technology. The research papers discuss the unique characteristics of novel soft materials, innovative techniques for aligning liquid crystals (LCs), and the integration of cells with LCs, as well as electro-optical and electrothermal effects, investigations of dielectric anisotropy, and dielectric spectroscopy, among other topics.

The paper by Cîrcu et al. [contribution 1] explores columnar liquid crystals of copper(I) complexes, showcasing the dual properties of ionic conductivity and solid-state emissions. The research demonstrates how benzoylthiourea-based ligands facilitate the formation of stable columnar phases with strong hydrogen-bonding networks. The new copper(I) halide complexes exhibit hexagonal columnar mesophases over a broad temperature range. In solid states, the new complexes show green emission while the luminescence properties are influenced by the nature of halide ions (chloride or bromide), with quantum yields reaching 8% at room temperature. In addition, the dielectric spectroscopy measurements indicated anhydrous proton conduction, a rare feature in metallomesogens, with a maximum conductivity of 1.37 × 10^−6^ S·cm^−1^ at 440 K for the bromide–copper(I) complex.

The design and synthesis of novel liquid crystalline materials are crucial for the advancement of innovative materials with enhanced characteristics. Thus, in their study, Urbańska et al. [contribution 2] report on the synthesis and characterization of novel chiral smectic esters compounds, emphasizing their potential in high-contrast electro-optic devices. These orthoconic antiferroelectric liquid crystals (OAFLCs) exhibit unique self-organizing properties [3]. The synthesized four-ring esters type compounds demonstrate an extensive *SmC_A_* phase range, an essential criterion for practical applications. Moreover, enhanced helical pitch and tilt angles were achieved without significantly altering spontaneous polarization. This research facilitates the advancement of OAFLCs mixtures, ensuring enhanced optical performance in high-speed display applications.

The study reported by Liu et al. [contribution 3] presents a novel approach to enhancing reflective bandwidths through a precisely engineered trilayer system. Cholesteric liquid crystals (CLCs) have long been recognized for their unique optical properties, particularly their ability to selectively reflect specific wavelengths of light [4]. The study introduces a groundbreaking photoinduced diffusion method that exploits the differences in polymerization rates of monofunctional and bifunctional polymerizable monomers. This technique results in a gradient pitch distribution, effectively broadening the reflective bandwidth to 1570 nm—a significant enhancement compared to traditional approaches. At the core of this advancement is the development of a trilayer cholesteric liquid crystal composite system. By leveraging hydrophilic and hydrophobic glass surface treatments, researchers were able to create a structured diffusion environment where polymer networks act as diffusion channels. This configuration facilitates controlled monomer movement, leading to an optimized pitch gradient and superior infrared reflection.

Tseng et al. [contribution 4] designed a novel double-cell cholesteric liquid crystal (CLC) device, capable of bicolor tuning and hyper-reflective color switching. Their research employs electrothermal modulation to achieve dynamic optical properties. This approach presents a low-voltage, high-reflectivity solution for display and photonic applications. The new device integrates both left-handed (LH) and right-handed (RH) CLC layers, exploiting pseudo-dielectric heating for asymmetric optical responses. Hyper-reflective states were obtained via stacked CLCs, with central wavelengths tunable between 720 nm at 23.4 °C and 380 nm at 29.8 °C.

D. Kostikov and al. [contribution 5] turned their attention to the study of chiral nematic LCs with tangential–conical boundary conditions under an electric field. Using the LN-396 cholesteric LC and the alignment polymer, PiBMA (poly(isobuthyl methacrylate)), a certain conical anchoring is assigned to the mentioned LC. The addition of poly(methylmethacrylate) (PMMA) or poly(tertbutyl methacrylate) (PtBMA) to PiBMA results in a decrease in the tilt angle inversely proportional to the polymer’s weight content. In the PiBMA/PMMA mixture, if the PMMA content is less than 60%, on the substrate with conical anchoring, the chiral nematic LC initially forms domains with tilt angles of the same magnitude but different signs. The authors used the rotating analyzer method to determine the azimuthal orientation for the defect-free structure, the structure containing linear defects, or the periodic structure. A transient azimuthal inhomogeneity is observed within the structure under voltage, independently of the PMMA content in the orienting PiBMA/PMMA films. It persists from ten to several hundred minutes. The memory-like structures are not formed on the PiBMA/PtBMA orienting coating. Consequently, the director is twisted by the larger angle using the polymer mixture with PtBMA in comparison with PMMA, with the effect being explained by the easier gliding of the director on the polymer PiBMA/PtBMA film. The authors concluded that when using the PtBMA film as alignment layer in cells, the director tilt angle is easily controlled due to the conical boundary conditions, and thus this alignment layer might improve electro-optical devices. Also, the obtained structures when using PMMA films possess memory and can be considered metastable states, and their lifetime depends on the orienting film composition and the value of the applied voltage; hence, the effect could be of potential use in electro-optical devices.

The work by Kocot et al. [contribution 6] provides critical insights into the formation of the twist–bend nematic (N_TB_) phase in liquid crystal dimers. The twist–bend nematic phase is an exotic liquid crystal phase that exhibits a spontaneous chiral helical structure despite being composed of achiral molecules [5]. It is characterized by a short-pitch helical arrangement, where the director (molecular orientation) follows a nanoscale periodic modulation in a bent and twisted configuration. This phase was first discovered in certain dimeric liquid crystal molecules with a flexible spacer between rigid mesogenic units. In this study, the authors used broadband dielectric spectroscopy to investigate the molecular dynamics of thioether-linked cyanobiphenyl dimers. The N_TB_ phase formation is modulated by longitudinal dipolar correlations, resulting in distinct relaxation processes. The authors observed a critical-like dynamic scaling behavior for the investigated materials, suggesting cooperative molecular motions approaching the glass transition temperature. This study advances the understanding of molecular relaxation mechanisms in complex liquid crystalline systems, with implications for next-generation electro-optic materials.

Lin et al. [contribution 7] reported on a new type of polymer-dispersed liquid crystal (PDLC) device. By introducing a fluorescent dye 7-amino-4-methylcoumarin (AMCA), an absorption peak in the UV range was observed and upon UV irradiation there is the possibility of blue fluorescence [6]; by varying the concentration of the fluorescent material, a reduction in saturation voltage and an increase in contrast ratio were obtained. The LC droplets are encapsulated, their size depending on the photo-polymerization intensity, determining in turn the driving voltage. By dividing the cell into two zones in which the initial mixtures composed of liquid crystal, polymer, monomer, photoinitiator, and fluorescent materials were introduced and by exposing these zones to different polymerization intensities, two domains were obtained on the same sample that transmit light differently, even if the same voltage is applied to the cell. Thus, the electro-optical transmission will vary in the sample, depending on the applied voltage and the observed domain, with three possibilities demonstrated: total scattering (opaque), semitransparent, and totally transparent.

Ben Salah et al. [contribution 8] investigated the physical properties of a novel series of hydrogen-bonded liquid crystals (HBLCs) derived from simple and fluorinated 4-n-alkoxybenzoic acids and 2-fluoro-4-nitrobenzoic acid (FNBA). The mesophase characterization was carried out using traditional methods: DSC (differential scanning calorimetry) and POM (polarizing optical microscopy) [7]. A PDLC film revealing a polystyrene matrix and a FNBA/9OBAF HBLC blend was obtained, where 9OBAF stands for 4-nonyloxy-3-fluorobenzoic. The study concluded that a fluorine substituent and the presence of a NO_2_ group in the molecule led to increased dielectric permittivity, DC conductivity, dielectric anisotropy, and birefringence. The enhancement in the permittivity and dielectric losses at low frequency were attributed to the ionic contributions and ionic diffusion. Moreover, the ion concentration and the diffusion coefficient were obtained by fitting the permittivity and the dielectric loss spectra, and thus, the ion mobility was calculated. This study is an illustration of using molecular manipulation to increase the physical properties that are useful in the applications of advanced materials.

## 3. Conclusions

The collective contributions of these studies underscore the versatility and tunability of liquid crystalline materials. From metallomesogens with dual luminescent and conductive properties to twist–bend nematic dynamics, hyper-reflective cholesteric structures, and advanced ferroelectric formulations, the field is witnessing significant strides toward functional, application-driven materials. Looking ahead, challenges remain in enhancing stability, optimizing electro-optic responses, and integrating liquid crystalline materials into scalable technologies. However, these studies reaffirm the interdisciplinary potential of liquid crystal research, bridging chemistry, physics, and materials science to fuel next-generation optoelectronic and photonic applications. The continued exploration of new molecular architectures and hybrid systems will be crucial for further expanding the capabilities of liquid crystalline materials in practical applications.
